# Costs and Cost-Effectiveness of mCME Version 2.0: An SMS-Based Continuing Medical Education Program for HIV Clinicians in Vietnam

**DOI:** 10.9745/GHSP-D-22-00008

**Published:** 2022-08-30

**Authors:** Lora L. Sabin, Aldina Mesic, Bao Ngoc Le, Nafisa Halim, Chi Thi Hue Cao, Rachael Bonawitz, Ha Viet Nguyen, Anna Larson, Tam Thi Thanh Nguyen, Anh Ngoc Le, Christopher J. Gill

**Affiliations:** a Department of Global Health, Boston University School of Public Health, Boston, MA, USA.; b Department of Global Health, University of Washington, Seattle, WA, USA.; c Consulting Research for Community Development, Hanoi, Vietnam.; d Vietnam Administration for AIDS Control, Ministry of Health, Hanoi, Vietnam.; e Center for Population Research Information and Databases, General Office for Population and Family Planning, Ministry of Health, Hanoi, Vietnam.; f Hanoi Medical University, Hanoi, Vietnam.

## Abstract

**Background:**

The Mobile Continuing Medical Education (mCME) 2.0 project was a randomized controlled trial that found that a 6-month text message-based CME intervention improved both the use of online medical training resources and medical knowledge among a cadre of HIV clinicians in Vietnam. This companion study analyzed intervention costs and cost-effectiveness.

**Methods:**

We conducted (1) a financial analysis based on costs incurred during the trial’s planning and implementation; (2) an economic analysis to consider resource utilization; and (3) cost-effectiveness analyses to estimate cost inputs relative to impact: increase in self-study (measured by visits to online courses) and increase in knowledge (measured by exam score improvement) (in 2016 US$). Finally, we estimated the economic cost of a 9-month national program and a 10-year scaled-up model (in 2021 US$).

**Results:**

The total financial cost of the intervention was US$49,552; the main cost drivers were personnel time (71.4%) and technology inputs (14.9%). The total economic cost was estimated at US$92,212, with the same key cost inputs (representing 77.7% and 8.0%, respectively, of total costs). The financial cost per 10% increase in accessing online courses was US$923, while the cost of improving knowledge, measured by a 10% improvement in mean exam score across the study population, was US$32,057 (US$605 per intervention clinician). The comparable total economic cost of each improvement, respectively, was US$1,770 and US$61,452 (US$1,159 per intervention clinician). A future 9-month national program was estimated to cost US$37,403, while the full 10-year scaled-up program was estimated at US$196,446.

**Conclusions:**

This analysis indicates that leveraging mobile technology could be a feasible way to provide distance learning to health professions across Vietnam at a relatively low cost. Given the need for practical ways to expand CME in resource-constrained regions of the world, this approach warrants further study and possible adoption.

## Abstract (precis)

This cost analysis found that a mobile phone-based continuing medical education (mCME) intervention, involving daily text messages with links to relevant materials, for HIV clinicians in northern Vietnam was relatively low-cost and cost-effective, particularly for future nationwide models. Such mobile approaches to CME are worthy of attention in resource-constrained settings.

## Teaser

**Key Findings:**

- This study found that a highly successful phone-based continuing medical education (CME) intervention for HIV clinicians in northern Vietnam was relatively low-cost and cost-effective when costs were compared to benefits.
- Forecasted future models that spread fixed costs over expanded nationwide versions of the program are even more appealing from a cost perspective.
- These findings suggest that mobile approaches to CME can be quite cost-effective and are worthy of attention in resource-constrained settings.

**Key Implications:**

- Policy makers and national stakeholders should consider alternatives to traditional in-person CME, including mobile approaches (based on text messages) that are convenient for participants, effective, and relatively low cost to implement.
- Policy makers in resource-constrained environments should consider investing in innovative and cost-effective mobile phone-based CME on a long-term and widespread basis given that such strategies may have considerable set-up costs but can motivate clinicians to improve self-study habits and increase knowledge in critical ways.

## INTRODUCTION

Continuing medical education (CME) has received increased attention due to the direct links between clinicians’ knowledge, their performance in the field, and patient outcomes.^1^^–^^6^ In high-income countries, CME is typically delivered via conferences, meetings, and high-quality online courses. In many low- and middle-income countries, there is growing interest in developing strategies to maintain knowledge and strengthen the skills of the medical workforce, though implementation of such strategies often flounders due to inadequate resources and competing needs. Researchers have called for more research on ways to provide CME in these settings, urging a focus on the effectiveness and cost-effectiveness of CME alternative delivery modes.^1^^,^^7^^–^^11^ These include learning modalities that make use of mHealth technologies and various online platforms.

Vietnam, a rapidly growing middle-income nation, is a prime example of a country that has committed to expanding CME. In 2009, Vietnam’s government passed a new law on medical examination and treatment (LET) mandating licensure of all clinicians, along with documented CME activities to retain licensure.^12^^,^^13^ Under the law, the Ministry of Health (MOH) in Vietnam provides training (including continued and refresher training) to medical clinicians, who, in turn, are required to complete 48 hours of CME every 2 years. Failure to comply can result in a medical license being revoked. Initial development and management of new online CME courses, databases, and monitoring systems were supported by major investments from the Asian Development Bank’s “Health Human Resources Sector Development Program” (2011–2015)^14^ and a US$106 million World Bank loan focused on improving medical education.^15^ In 2020, a National Medical Council was established to guide and regulate medical training standards, licensing requirements, and preparation for national licensing examinations (Bao Ngoc Le, MA, CRCD, written communication, December 2021). Additionally, an amended version of the LET, which strengthens key LET provisions and requires relicensing every 5 years, awaits approval by the National Assembly as of April 2022.^16^

Since the LET’s passage, the MOH has experimented with ways to expand CME, including using novel strategies that take advantage of expanding the use of mHealth approaches. Such interest was driven largely by recognition of the expense and inconvenience of traditional, in-person CME modalities, which often also left medical facilities short of staff (Bao Ngoc Le, MA, CRCD, written communication, May 2022). As part of this effort, 2 mobile phone-based CME (mCME) approaches were tested by Boston-based and local Vietnam government and nongovernment researchers. The first version involved implementation of a 6-month randomized controlled trial (RCT) in 2015–2016 that assessed the delivery of daily short message service (SMS, or text) messages containing quiz questions of medical facts that were designed to pique interest in self-study among a large cadre of community-based physicians’ assistants in Thai Nguyen province. While feasible and widely accepted, the intervention failed to improve knowledge among participants.^17^

The Vietnam MOH has tested mobile phone-based approaches to CME, recognizing the expense and inconvenience of in-person approaches.

Based on qualitative data collected from participants that suggested shortfalls in the initial approach,^18^ the research team enhanced features of the intervention and reassessed it in a second 6-month RCT in 2016–2017 (mCME v2.0) (Box). Briefly, the mCME v2.0 approach was used among HIV physicians and other HIV care providers in 3 northern provinces of Vietnam.^19^ The study aimed to increase motivation for self-study and improvement in HIV-related medical knowledge. The mCME v2.0 intervention proved to be successful in changing study habits as well as improving medical knowledge.^19^ It was also extremely popular with participants.^20^^–^^22^

BOX **Overview of the Mobile Phone-Based Continuing Medical Education v2.0 Randomized Control Trial**

The mobile phone-based continuing medical education (mCME v2.0) intervention was a randomized control trial (registered on clinicaltrials.gov as NCT02381743) conducted from November 2016 to May 2017 in 3 northern provinces in Vietnam: Thai Nguyen, Quang Ninh, and Hai Phong. The 3 provinces differ geographically and represent varying levels of economic development. Thai Nguyen is less developed, largely rural, remote, and mountainous area (with a high proportion of ethnic populations and remote villages). Quang Ninh is a diverse region geographically, with a range of mountains, seacoast, islands, and lowlands, that is developing rapidly in urban areas. Hai Phong, and particularly Hai Phong City, is a more economically developed, industrial region, with a dense population.

To conduct the study, Boston University researchers partnered with Ministry of Health partners at the Center for Population Research Information and Databases, a subdivision of the General Office for Population and Family Planning, and Vietnam Administration for AIDS Control. The primary local academic partners were Hanoi Medical University and the Hanoi University of Public Health, with daily implementation carried out by collaborators at the Vietnamese nongovernmental organization, Consulting, Researching on Community Development, who had also played an important role in the first mCME study (2015–2016).

Of the 120 practitioners engaged in HIV care in the provinces and invited to participate in an initial 100-question exam, a total of 106 clinicians were randomized into either (1) the intervention group (n=53), which received daily short message service (SMS) quiz questions organized into HIV-related themes based on Vietnam’s national guidelines for care and treatment of individuals living with HIV, a follow-up SMS to indicate correct/incorrect response, an SMS with links to readings and an online course relevant to the theme, and a feedback SMS with the individual’s performance compared to the rest of the group; or (2) the control group (n=53), which received a weekly non-medical SMS and access to the online courses but no quiz questions, readings, or feedback. Assessment of intervention impact was done via data gathered automatically from the online course website, which tracked clicks to the online courses but not course-specific exam results, scores on a second 100-question exam at the 6-month study endpoint (for a total of 95 clinicians, 92.2% of all those enrolled), and data collected through a survey of clinicians that asked about study habits. Additional details on study procedures as well as intervention results have been published previously.^19^^–^^22^

The intervention group was significantly more likely to access the courses (134 times versus 27 times, *P*<.02) and more likely to ever use the online courses (60.4% versus 26.4%, respectively, relative risk: 2.3, 95% confidence interval [CI]=1.4, 3.8). While both groups displayed an increase in knowledge per score on a 100-question exam (over a 6-month period), the percent change increase was 13 percentage points higher in the intervention group than in the control group (mean: 26% (95% CI=16, 35) versus 13% (5, 22) respectively, *P*=.06).^19^

The RCT was unable to assess an accompanying improvement in clinical skills or patient outcomes, as this would have required a much larger and complex study design. Indeed, to show such an effect, thousands of patients would need to be followed for a sufficient amount of time for the health benefits to accrue. That also is challenging in a study such as the mCME trial, where the process outcomes, study behaviors, and knowledge are not the same as a patient health outcome. However, there is every reason to feel confident that clinical skills improved, given studies showing that HIV patients who are managed by clinicians with greater knowledge and expertise receive more appropriate care.^23^^–^^34^

Decisions on expansion and scale-up of novel interventions must be guided not just on benefit but also on cost. Yet, the published literature on economic evaluations of mHealth interventions generally is sparse.^35^^–^^37^ To add to our understanding of the costs relative to benefits of the mCME approach, both for future scale-up in Vietnam and implementation in other settings where mCME might be practical, we conducted a secondary cost analysis of the mCME v2.0 project. This encompassed multiple analyses, including a financial analysis of the actual costs incurred while planning and implementing the mCME intervention, and an economic analysis to better understand the full range of resources utilized. Additionally, to inform policy makers considering future scale-up of such an intervention (outside of the context of a research-based RCT), we also conducted an economic analysis of a future model expanded to national scale.

## OVERVIEW OF COST ANALYSIS

We conducted several cost analyses for the present study (Table 1). First, we analyzed the financial costs incurred during the planning and implementation of mCME v2.0. Second, we conducted an economic analysis, which better reflects the full resource costs of an intervention.^38^ Next, we compared costs and benefits of the intervention through cost-effectiveness analyses (CEA), both financial and economic, to inform stakeholders and policy makers about the cost of marginal effects attributed to the intervention. Finally, to contribute evidence for decision making, we estimated the economic cost of national scale-up of mCME v2.0 for HIV clinicians through both a 9-month program and a 10-year program.

**TABLE 1.** Overview of Cost Analyses of mCME v2.0 Program for HIV Clinicians in Vietnam

**Cost Analysis**	**Description**
Financial analysis	An analysis calculating the total financial expenditures associated with the 13-month research study.
Economic analysis	An analysis of the total economic costs (i.e., resource costs) for the 13-month research study, including volunteer time, travel not paid for by the study, and pre-developed infrastructure that was required for the intervention (i.e., online courses).
Financial cost-effectiveness analysis	An analysis calculating the incremental financial cost-effectiveness ratio, which was estimated by the difference in total financial costs between intervention and control groups, respectively, divided by the change in online course visits and endline exam scores for clinicians in the intervention and control groups, respectively.
Economic cost-effectiveness analysis	An analysis calculating the incremental economic cost-effectiveness ratio, which was estimated by the difference in total economic costs between intervention and control groups, respectively, divided by the change in online course visits and endline exam scores for clinicians in the intervention and control groups, respectively.
Forecasted analysis	An analysis designed to assist policy making related to CME: this involved estimating the costs of a future scaled-up program to all HIV clinicians in Vietnam (N=865). We estimated economic costs over 2 alternative time frames: a 9-month period (January 2021 to September 2021) and a 10-year period (January 2021 to January 2031).

Abbreviations: CME, continuing medical education; mCME, SMS-based CME.

### Financial Cost Analysis

The financial analysis of the intervention was based on expenditures associated with the full 13-month study, including 7 months of start-up activities from April 2016 to October 2016 and 6 months of intervention implementation from November 2016 to April 2017. We included the cost of personnel time for start-up (for both U.S. and Vietnam-based collaborators), project coordination (U.S. and Vietnam-based staff), and intervention monitoring and supervision (Vietnam-based staff). All incremental costs associated with development of the intervention text messages were included; thus, while both intervention and control groups received text messages, we included the marginal cost of developing the more complex messaging for the intervention group relative to the control group (estimated at 90% of total message development cost, based on relative time spent by the content developer). Additional start-up costs included accommodation, travel, and food associated with initiation of the program.

During the intervention phase, given that both groups received text messages and had access to online materials, we estimated the proportion of total costs represented by intervention group versus the control. As such, we estimated that the intervention group cost was 90% of the total, whereas the control group costs accounted for 10% of the total (i.e., cost of the text messages once a week, and a small proportion of technology costs, which included server maintenance and sending the text messages). The salaries for the clinicians were not paid by study funds and thus were excluded from the analyses. The time clinicians spent responding to texts and accessing online courses was also excluded. Our rationale was that self-study in CME was an expected and ongoing part of their job responsibility (underscored by relatively high reported rates of CME engagement at baseline)^19^ and responding to text messages and engaging in self-study took place almost entirely during off-work hours (and thus did not entail time away from work).^21^ Additionally, per the usual convention, all research costs were excluded. This included the development and implementation of the baseline and endline exams (6 months later), which were assessment tools rather than intervention components.

Because the mCME v2.0 program was an extension of a previous RCT,^17^ the technology infrastructure to deliver the text messages was transferred from the original trial, representing a key precondition to implementing mCME v2.0.^19^ This infrastructure, housed at the Center for Population Research Information and Databases (CPRID) at the MOH, was relatively simple, encompassing a server, a computer, and a SIM card to connect to the phone system in northern Vietnam. For the first trial, CPRID technicians had developed the software to send text messages to participants and record their responses. The cost of their time to develop this software was included in the financial analysis.

We converted nominal Vietnam-based costs into U.S. dollar equivalents using the average annual exchange rate for the year in which the costs were incurred^39^ and added them to the nominal U.S. dollar-based costs. We then adjusted total annual U.S. dollar costs by U.S. inflation rates (Consumer Price Index),^40^ and, as recommended by health experts (including the World Health Organization (WHO)),^38^^,^^41^ applied a discount rate of 3%. Program costs were thus expressed in real 2016 U.S. dollar figures. Costs were categorized as follows: personnel; travel, food and accommodation; technology; overhead. Within these categories, there were sub-categories for each partner (Boston University School of Public Health, CPRID, Hanoi Medical University, consultants, VAAC), each of which was organized into project stage: preparation and planning; or implementation and management. We also categorized fixed costs unrelated to the number of participating clinicians or the number of courses offered (e.g., preparation and planning costs, program implementation and management costs, and technology costs) versus variable costs (SMS costs and online course costs).

### Economic Analysis

Based on WHO recommendations, we conducted an economic cost analysis of the intervention which provides more accurate estimates of the true economic resource costs of an intervention than financial costs alone.^42^ First, we included volunteer personnel time used for program planning, implementation, and data collection. This was calculated by multiplying the salaries and time spent working on the project of each of the investigators. Next, we included travel, accommodation, and food costs that had been incurred privately and not as part of project expenses. We also included the cost of the online courses, which were developed before the current trial but represented critical infrastructure for the intervention and, therefore, an important element for the economic analysis. Relatedly, we calculated overhead based on the total economic direct costs. As with the financial analysis, all U.S. dollar nominal costs were adjusted for U.S. inflation rates, discounted at 3%, and expressed in real 2016 U.S. dollars.

### Cost-Effectiveness Analyses

The key outcome used for the CEA was the primary outcome measure of the trial: the difference in accessing the online courses between intervention vs. control groups. The intention to treat analysis found that intervention clinicians visited the online courses 134 times, compared to a total of 27 visits among the control group (or 486% higher). We used increments of 10% for the CEA. We conducted an additional CEA based on a secondary outcome, which was improvement in HIV knowledge among clinician participants. For this outcome, the intention to treat analysis found a difference in knowledge improvement (from baseline exam to endline exam) between intervention and control clinicians of 26% versus 13%, or 13%. Similar to the CEA for the primary outcome, we assessed the cost of a 10% increase in knowledge score between groups. In each case, we used the following formula:

$${\text{ICFA}=(C}_{1}-C_{C})/{(O}_{1}-O_{C})$$

where ICEA is the incremental cost-effectiveness ratio; C_I_ and C_C_ are the total (discounted) costs associated with intervention and control groups, respectively; and O_I_ − O_C_ are the (discounted) change in online course visits and endline exam scores for clinicians in the intervention and control groups, respectively.

### Forecasted Program Analyses

For the forecasted analysis, we estimated the costs of a future program to scale up mCME to all HIV clinicians in Vietnam, estimated at n=865 (Chi Thi Hue Cao, PhD, VAAC, written communication, July 2018). We estimated economic costs over 2 alternative time frames: a 9-month period (January 2021 to September 2021) and a 10-year period (January 2021 to January 2031). The 9-month program encompassed a single program consisting of 3 months of preparation and 6 months of text messaging (like the trial), while the 10-year model included an initial 9-month program in Year 1 as well as delivery of the 9-month program at 4 additional time points in Years 3, 5, 7, and 9 (Table 2). The 10-year program would meet the national mCME requirement that clinicians must complete 48 hours of CME every 2 years (failure to comply can result in revocation of the clinician’s medical license).^1^ The forecasted analysis was limited to costing only, as it is impossible to say with any certainty that a future program would improve online course use or knowledge by any specific extent.

**TABLE 2.** Input Parameters for Univariate Sensitivity and Policy-Determined Variables for the Economic Forecasted Analysis of mCME v2.0 for HIV Clinicians in Vietnam

**Input Parameters for Univariate Sensitivity**	**Best Estimate**	**Minimum** ^a^	**Maximum** ^a^	**Source**
Number of clinicians	865	649	1,082	In-country partners^b^
Program management salaries (2021 US$ per month)	1000.00	750.00	1,250.00	In-country partners^b^
Consultancy fee, 2021 US$ per hour	50.00	37.5.00	625.00	In-country partners^b^
Technology (server, telephone, SMS system) costs, 2021 US$ per month	1,110.64	832.98	1,388.29	Trial^c^
**Policy-Determined Variables**	**9-month expansion,**^d^ **No.**		**10-year expansion,**^e^ **No.**	
Total months of preparation	3		12	
Total months of implementation	6		24	
HIV expert days for content refining	10		40	

Abbreviations: mCME, SMS-based continuing medical education; SMS, short message service.

a Sensitivity values are -25% of the best estimate for the lower bound and +25% of the best estimate for the upper bound.

b In-country partners provided the best estimates for the cost of labor (i.e. program management salaries; the consultancy fees) and the number of HIV clinicians in-country for whom this program could be scaled up.

c Costs for technology were estimated based on costs during the trial with considerations for inflation.

d The 9-month expansion includes shifting all labor costs to local staff for 3 months of preparation (including 10 days of an HIV expert to refine content), and a 6-month intervention.

e The 10-year expansion includes the costs from the 9-month expansion in year 1, and then additional preparation/implementation in years 3, 5, 7, and 9.

Cost projections for the scaled-up version were primarily associated with set-up, personnel, and infrastructure. Regarding set-up, we assumed the preexistence of the SMS system and online courses. Preparation thus focused on updating existing materials and obtaining contact information on all HIV clinicians. Personnel costs incorporated the salaries of local staff, based on the experience of the mCME v2.0 trial but including shifting of responsibilities from Boston-based collaborators to local personnel: 2 full-time staff members from CPRID for essential planning and implementation of text messages and 10 days of time for an HIV consultant to refine course and SMS content during each preparation period. Future salary estimates for the base year (2021) were based on in-country conservative estimates. No Boston-based personnel were included in program planning and implementation. All costs for food, accommodation, and travel were excluded from the scale-up as these costs were minimal during the trial and largely incurred by out-of-country partners that were no longer part of the scale-up.

For technology costs, we assumed that the costs for maintaining the server, telephone line, and SMS system would be the same as the costs incurred during the trial. For the cost of text messages, we estimated 3 text messages per day over a 6-month intervention for all clinicians in country. We also included a sensitivity analysis (+/−25%) for the salary parameters (both CPRID personnel and consultants) and for the technology costs.

Given that the intervention was planned to begin in the next year, we inflated the costs for both the 9-month national expansion and the 10-year forecasted analysis based on average inflation over the most recent 5 years in Vietnam (2.64%).^43^ To estimate the increases in salary over time, we relied on estimates from partners in country (Bao Ngoc Le, MA, CRCD, personal communication, June 2020), assumed similar indirect costs as during the trial, and discounted all costs by 3%, similar to the other analyses. Table 2 includes input parameters for univariate sensitivity analysis and the policy-determined variables.

### Ethics Approval

The original trial was approved by the ethical committees at Boston University Medical Center (Boston, MA) and The Hanoi School of Public Health (Hanoi, Vietnam). All clinicians that participated in the RCT gave their written informed consent. We did not obtain separate informed consent for the present study because we used only de-identified, aggregated outcome data from the original trial.

## RESULTS

### Financial and Economic Costs

Financial and economic costs are presented according to major cost item (personnel, travel/food/accommodation, technology), partner (Boston-based or Vietnam-based partners), and program period (preparation versus implementation) (Table 3). The total financial costs were US$49,553 (2016 U.S. dollars), and the main cost items were personnel time and technology.

**TABLE 3.** Financial and Economic Costs of mCME 2.0 Intervention for HIV Clinicians in Vietnam^a^^,^^b^

	**Financial Cost, US$**	**% of Total**	**Economic Cost,^c^ US$**	**% of Total **
Personnel				
BUSPH				
Preparation and planning^d^	6,081.44	12.3%	16,545.11	17.9%
Program implementation and management^e^	13,016.13	26.3%	18,965.36	20.6%
CPRID				
Preparation and planning	1,591.30	3.2%	8,936.46	9.7%
CRCD				
Preparation and planning	2,803.17	5.7%	2,803.17	3.0%
Program implementation and management	3,181.19	6.4%	3,181.19	3.4%
HMU				
Preparation and planning	0	0%	12,516.91	13.6%
Consultants^f^				
Preparation and planning	3,417.66	6.9%	3,417.66	3.7%
VAAC				
Preparation and planning	5,297.32	10.7%	5,297.32	5.7%
Total personnel	35,388.21	71.4%	71,663.19	77.7%
Travel, Food, and Accommodation				
BUSPH				
Program implementation and management	264.64	0.5%	264.64	0.3%
CPRID				
Preparation and planning	0	0%	541.31	0.6%
VAAC				
Preparation and planning	247.84	0.5%	247.84	0.3%
Total travel, food, and accommodation	512.48	1.0%	1,053.79	1.1%
Technology^g^				
CPRID				
Preparation and planning	7,362.09	14.9%	7,362.09	8.0%
Total technology	7,362.09	14.9%	7,362.09	8.0%
Overhead				
Indirects	6,289.98	12.7%	12,133.05	13.2%
Total indirects	6,289.98	12.7%	12,133.05	13.2%
Total				
Total cost (discounted)	49,552.76	100.0%	92,212.12	100.0%
Total cost for preparation period	26,184.66	52.8%	61,313.95	66.5%
Total cost for implementation period	23,368.10	47.2%	30,898.17	33.5%

Abbreviations: BUSPH, Boston University School of Public Health; CPRID, Center for Population Research Information and Databases; CRCD, Consulting, Researching on Community Development; HMU, Hanoi Medical University; mCME, mobile continuing medical education; SMS, short message service; VAAC, Vietnam Administration for AIDS Control.

a All figures are presented in real 2016 US$.

b All costs are discounted by 3%.

c The economic analysis considers the actual time spent on the project by personnel, as this project required a substantial amount of volunteer time from BUSPH staff. The economic analysis took into consideration the costs of the online courses developed by HMU and the cost of the SMS system from mCME 1.0.^17^

d Preparation and planning refers to costs during the prep period for the trial from April 1, 2016 to October 30, 2016.

e Program implementation and management refers to costs during the implementation period for the trial from November 1, 2016 to April 30, 2017.

f Consultants were used to complete the following tasks: link SMS system with HMU, test functionality of SMS system, and develop content and review translation.

g Technology includes the cost of maintaining the server, the SMS messages to the intervention and control groups, and the monthly fee for the SMS system.

For nearly every category except for personnel, the financial and economic costs were very similar. This project required a substantial amount of volunteer time from Boston University School of Public Health staff, which was captured in the economic analysis for a total of US$92,212. The total economic cost of personnel was US$71,633, representing 77.7% of total economic cost.

Table 4 provides the total financial and economic costs by fixed and variable costs. Most costs were fixed, namely, preparation and planning during the start-up phase (38.0% of the total) and program management during implementation (nearly 47.2% of the total). Technology costs accounted for 13.4% of total costs. Variable costs were mainly the costs of the technology (1.5%), which included the cost of the server for courses and sending text messages. In the economic cost analysis, fixed costs also accounted for most of the total cost but were slightly less due to the influence of the addition of the online courses, which were developed as part of the previous intervention (13.6% of total economic cost).

**TABLE 4.** Fixed and Variable Costs of the mCME 2.0 Intervention for HIV Clinicians in Vietnam^a^

	**Financial Cost**		**Economic Cost**	
	**(April 2016–April 2017)**		**(April 2016–April 2017)**	
	**Cost, US$**	**% of Total**	**Cost, US$**	**% of Total**
Total fixed costs^b^				
Preparation and planning^c^	18,822.57	37.98%	41,434.95	44.93%
Program management^d^	23,368.10	47.16%	30,898.17	33.51%
Technology (server, telephone line, SMS system)	6,633.81	13.39%	6,633.81	7.19%
Total variable costs^e^				
SMS^f^	728.28	1.47%	728.28	0.79%
Courses^g^	0	0.00%	12,516.91	13.57%
Total costs				
Total costs	49,552.76		92,212.12	
Total fixed costs	48,824.48		78,966.93	
Total variable costs	728.28		13,245.19	

Abbreviations: mCME, mobile continuing medical education; SMS, short message service.

a All figures are presented in real 2016 U.S. dollars.

b Fixed costs included program preparation and planning, program management, technology (i.e., SMS system), and travel and food/accommodation related to program preparation and planning and program management.

c Preparation and planning refers to costs during the prep period for the trial from April 1, 2016 to October 30, 2016.

d Program implementation and management refers to costs during the implementation period for the trial from November 1, 2016 to April 30, 2017.

e Variable costs included the number of text messages, which would vary depending on the number of clinicians in the program, and the number of courses, which would vary depending on the course topic. For mCME 2.0, there were 12 courses focused on HIV/AIDS.^19^

f Each SMS was US$0.025. The control group was sent one SMS per week, and the intervention group received 3 SMS every day during the 6-month intervention.

g Cost of online course development is based on information provided by in-country partners.

### Financial and Economic Cost-Effectiveness

The results of the cost-effectiveness analysis are shown in Tables 5 and 6. The financial cost-effectiveness of the key outcome variable, a 10% increase in online course visits by clinicians, was US$923, while the economic cost-effectiveness was slightly higher, at US$1,770 (Tables 5 and 6). The financial cost-effectiveness of a 10% increase in knowledge (measured by mean change in exam scores) was US$32,057 for the full intervention study population. This corresponds to US$605 per clinician in the intervention group. The economic cost-effectiveness was higher, at US$61,452 total, or US$1,159 per clinician (Tables 5 and 6).

**TABLE 5.** Financial Cost-Effectiveness of mCME 2.0 Intervention for HIV Clinicians in Vietnam^a^

Cost-effectiveness per 10% increase in online course visits^b^^,^^c^	US$923.44
Cost-effectiveness per 10% increase in endline knowledge score^b^^,^^d^	US$32,056.53
Cost per intervention participant	US$890.87
Cost per control participant	US$44.09

a All figures are presented in real 2016 U.S. dollars.

b For the intervention group, the cost was the full cost of the preparation period and 90% of the intervention costs (US$47,215.95 total). For the control group, the costs consisted of 10% of the implementation costs (i.e., cost of the text messages once a week, and a small amount of the cost to maintain the server and SMS system for a total of US$2,336.81).

c Clinicians in the intervention group visited the online courses 134 times, and clinicians in the control group visited the online courses 27 times. Overall, the difference in online course visits between the intervention and control group was 107, which is a 486% increase. The following equation was used to determine cost-effectiveness: ICEA = (CI−CC)/(MI−MC), where ICEA is the incremental cost-effectiveness ratio; CI and CC are the total (discounted) costs related to the intervention and control groups, respectively; and MI−MC are the (discounted) change in online course visits. Therefore, the equation was as follows: (US$47,215.95–US$2,336.81)/(486%)=US$92.343 for a 1% increase (or *10=US$923.44 for a 10% increase).

d The average change in score for the intervention group was +26% between baseline and endline exams. The average change in score for the control group was +12%. The following equation was used to determine cost-effectiveness: ICEA = (CI−CC)/(MI−MC), where ICEA is the incremental cost-effectiveness ratio; CI and CC are the total (discounted) costs related to the intervention and control groups, respectively; and MI−MC are the (discounted) percent change in exam scores. Therefore, the equation was as follows (US$47,215.95–US$2,336.81)/(26%-12%)=US$3,205.65 per 1% increase, so US$32,056.53 for a 10% increase in knowledge for the full study population.

**TABLE 6.** Economic Cost-Effectiveness^a^

Cost-effectiveness per 10% increase in online course visits^b^^,^^c^	US$1,770.21
Cost-effectiveness per 10% increase in end line knowledge score^b^^,^^d^	US$61,451.77
Cost per intervention participant	US$1681.55
Cost per control participant	US$58.30

a All figures are presented in real 2016 U.S. dollars.

b For the intervention group, the cost was the full cost of the preparation period and 90% of the intervention costs (US$89,122.30). For the control group, the costs consisted of 10% of the implementation costs (i.e., cost of the text messages once a week and a small amount of the cost to maintain the server and SMS system, US$3089.82).

c Clinicians in the intervention group visited the online courses 134 times, and clinicians in the control group visited the online courses 27 times. Overall, the difference in online course visits between the intervention and control group was 107. The following equation was used to determine cost-effectiveness: ICEA = (CI−CC)/(MI−MC), where ICEA is the incremental cost-effectiveness ratio; CI and CC are the total (discounted) costs related to the intervention and control groups, respectively; and MI−MC are the (discounted) change in online course visits. Given that we wanted to calculate the percentage increase, the percentage increase from 27 visits to 134 is 486%. Therefore, the equation was as follows (US$89,122.30–US$3089.82)/(486%)=177.02 for a 1% increase or *10 = US$1770.21 for a 10% increase.

d The average change in score for the intervention group was +26% between baseline and endline. The average change in score for the control group was +12%. The following equation was used to determine cost-effectiveness: ICEA = (CI−CC)/(MI−MC), where ICEA is the incremental cost-effectiveness ratio; CI and CC are the total (discounted) costs related to the intervention and control groups, respectively; and MI−MC are the (discounted) change in exam scores. Therefore, the equation was as follows (US$89,122.30–US$3089.82)/(26%–12%) = US$6,145.12 for a 1% increase or *10=US$61,451.77 for a 10% increase in knowledge for the full study population (N=53).

### Forecasted Program: Economic Costs

Table 2 presents the input parameters for the forecasted cost analysis, and Table 7 presents the results. A 9-month expansion of the program from January 2021 to September 2021 would cost an estimated US$37,403, which includes local rather than international labor, 3 months of preparation and planning, 10 days of an HIV expert’s time, technology costs for 6 months, and the variable costs for additional text messages for all HIV clinicians in country. As expected, the majority of costs were related to personnel (US$18,702) followed by technology costs (US$6,924). When using the lower bound estimates (i.e., -25% best estimate for personnel, consultant fees, number of clinicians, and technology costs), the total costs was estimated to be US$27,579, whereas when using upper bound estimates (i.e., +25% best estimate for personnel, consultant fees, number of clinicians, and technology costs), the total cost was estimated to be US$44,420 (Table 7).

**TABLE 7.** Scale-up by Cost Category for 9-Month and 10-Year Scenarios (January 2021–September 2021)^a^

	**Base Case,**^c^ **US$**	**Optimistic,**^d^ **US$**	**Conservative,**^e^ **US$ **
**9-Month Scale-up Costs by Category** ^b^			
Project management personnel	18,702.00	14,026.50	23,377.50
Consultants	4,156.00	3,117.00	5,195.00
Technology costs (server, telephone line, SMS system)	6,923.70	5,192.77	8,654.62
SMS messages	2,050.46	2,050.46	2050.46
Overhead	4,329.79	3,192.57	5,141.98
Total costs	37,403.41	27,579.30	44,419.57
**10-Year Scale-up Costs by Category** ^f^			
Project management personnel	98,571.52	73,928.64	123,214.40
Consultants	21,904.78	16,428.59	27,380.97
Technology costs (server, telephone line, SMS system)	36,492.32	27,369.24	45,615.41
SMS	10,807.26	10,807.26	10,807.26
Overhead	22,126.74	16,826.86	27,101.56
Total costs	196,445.88	145,360.59	234,119.60

Abbreviations: SMS, short message service.

a These figures are presented in real 2021 U.S. dollars, discounted by 3%.

b The base case includes the best estimates for each of the categories.

c The optimistic scenario includes the lower bound estimates (i.e., -25% the best estimate) for program management personnel salary costs, consultant fees, and technology costs.

d The conservative scenario includes the upper bound estimates (i.e., +25% the best estimate) for program management personnel salary costs, consultant fees, and technology costs.

e The 9-month expansion includes shifting all labor costs to local staff for 3 months of preparation (including 10 days of an HIV expert to refine content) and a 6-month intervention.

f The 10-year expansion includes the costs from the 9-month expansion during year 1 and then additional preparation/implementation in years 3, 5, 7, and 9.

The majority of costs related to personnel and technology.

For the 10-year program from January 2021 to January 2031, the total cost is estimated to be US$196,446. This includes the full costs from the 9-month expansion, 9 additional months of preparation, 24 additional months of implementation, local staff costs for 36 months of work, technology costs for 24 implementation months, and variable costs for text messages for all clinicians for 24 months of implementation over the remaining 9 years. The lower bound estimate was US$145,361 whereas the upper bound was US$234,120 (Table 7).

## DISCUSSION

In this study, we quantified and described the financial and economic costs of development and implementation of a mCME program for HIV clinicians in Vietnam. This project began with the notable main study results, which indicated that an mCME intervention, relying on daily text messages to motivate self-learning, led to a significant increase in self-study behaviors among HIV clinicians in Vietnam, as measured by the number of visits to relevant online CME courses, and by an improvement in scores on a medical exam. To our knowledge, this study was the first to use an mHealth intervention in a resource-constrained setting to promote CME across a range of HIV topics. The costing and cost-effectiveness analysis in this article was designed to provide policy-relevant information for Vietnam and other regions where in-person CME faces substantial barriers and where a mCME approach may be useful.

This analysis indicated that providing mCME to HIV clinicians may represent a reasonable use of resources given the positive impact of the program on online course visits and medical knowledge among the clinicians who participated. While the total financial cost of the program was not minimal, at US$49,553, this cost is best viewed in the context of what it achieved. The program yielded an enormous increase (nearly 500%) in use of online courses designed specifically for CME among HIV clinicians. The Vietnamese government had invested in these courses as part of its commitment to strengthening CME and hoped they would prove to be a useful resource as the country sought to expand CME for HIV clinicians (Chi Thi Hue Cao, PhD, personal communication, July 2017). Yet, as the main RCT findings revealed, actual use among control group clinicians was quite low, raising doubts that the courses would serve a meaningful purpose in the absence of an intervention or requirement to use them. Importantly, these courses satisfy LET licensure requirements. Thus, our finding that a 10% increase in visits to the online courses correlated to an economic resource cost of US$1,770 (and a much lower financial cost of US$923) is significant and suggests that text messages to motivate use of online courses represents an efficient use of resources. This is particularly true when considering that the trial measured visits to the online courses over a 6-month time frame, and variable costs of the program were low—highlighting the fact that longer term use would likely further reduce the cost of increasing course visits.

Our analysis indicated that providing mCME to HIV clinicians may represent a reasonable use of resources given the positive impact of the program on online course visits and medical knowledge among the clinicians who participated.

The cost to benefit comparison of the mCME approach may be even more striking when considering increases in medical knowledge. Here, we found that the economic cost for a 10% increase in mean knowledge (measured by exam scores) among clinicians was US$61,452 (US$32,057 in financial terms). While not a small number, it is important to consider that this cost represents US$1,159 (US$605 in financial terms) on average for each of the participating clinicians in the trial. We are not aware of similar studies with which to compare these figures, but other studies have highlighted the value of CME in HIV care,^44^ the challenges in implementing such programs in different settings,^45^^,^^46^ and the rising need for point-of-care training for medical professionals globally to strengthen care in HIV and TB.^47^

We also note that although it was not possible to link definitively the changes in behavior we observed in the trial to specific practical behaviors in clinical practice, such changes are likely given the link between CME and clinical care in a variety of settings.^23^^–^^34^ Similarly, there would be clear benefit in showing that CME leads to improved patient outcomes, but this would require 2 things that are challenging to obtain. First is the time needed to measure the cumulative impact of education on medical skills, which one can predict would take years to manifest (not a realistic goal for a 6-month intervention). Second is the scale required of a trial designed to demonstrate the consequence of many individual pieces of knowledge and experience on outcomes such as CD4-cell counts or lives saved—many thousands of patients followed for a sufficient amount of time for the health benefits to accrue. Indeed, we are not aware of any large-scale attempt to link traditional CME activities in the United States, for example, to patient outcomes. Rather, CME is understood as being essential in this sense: if a clinician lacks knowledge, then their skill will obviously be reduced. But having such skill does not immediately or necessarily translate into concrete outcomes.

Our detailed cost analysis highlighted substantial fixed costs related to the mCME intervention (e.g., program planning, management, and technology inputs encompassing hardware and software development). These costs may be viewed as a critical investment by a government that has mandated CME requirements. At the same time, it is important to note that many of these costs could be modified and reduced were the program implemented over a longer time frame, rather than just the limited 13-month program we evaluated. Much of the required infrastructure for such a program (i.e., text message development, online course development) has been developed and can be tapped relatively easily for scale-up of a similar mCME program, as we illustrate in the 2 (9-month and 10-year) nationwide scale-up models. This infrastructure could also be used for similar programs across a range of health-related domains. Naturally, this effort would involve creating appropriate online courses and text messages, but the basic software infrastructure and local learning is in place to allow further expansions in mCME in HIV care as well as other medical fields without major investments.

Although our detailed cost analysis showed substantial fixed costs, many of these costs could be reduced by using existing infrastructure and implementing the program for a longer period.

Fundamentally, the mCME 2.0 trial was motivated by a need to test alternatives to expensive, cumbersome, and inaccessible in-person CME in settings where governments are attempting to strengthen CME systems and mobile technologies represent promising and flexible tools. As noted previously, Vietnam has committed to expanding CME requirements and to providing relevant programs, yet actual expansion of feasible CME is challenging due to the cost and inconvenience for providers. We understand from study partners that alternatives such as attendance at scientific conferences may be allowed to meet CME obligations, though such options would be challenging wherever travel is necessary (and this would not solve the problem of having to replace providers who are away from their posts), in addition to online course opportunities (Bao Ngoc Le, MA, written communication, May 2022). Here, it may be worth considering how costs might compare between the mCME approach and a more traditional in-person CME. Comparing a daily text message program designed to spur self-study and an in-person training designed to increase knowledge over a 1- or 2-day period is admittedly problematic. We are unaware of published data on the latter. However, discussions with our in-country partners suggest that providing an in-person 2-day training to HIV providers might be estimated at US$10,000–US$12,000 for 100 participants (including costs of venue, training materials, travel, accommodation, and food) or close to US$100,000 for all HIV providers nationwide (n=865 in our forecasted analysis) (Bao Ngoc Le, MA, written communication, May 2022). This cost would likely be lower in rural areas and 30%–40% higher in urban centers such as Hanoi and Ho Chi Minh City. While not an exact comparison, this level of cost compares favorably with our estimate of approximately US$37,000 for implementing a mCME program such as the one tested in our trial.

At this point, we highlight important advantages of the approach we tested in the mCME v2.0 trial above and beyond the costs measured here. The general approach was well-liked by participants, who noted they found daily texts motivating and relevant.^18^^,^^21^ It also has the advantage of allowing medical personnel to remain in their positions rather than requiring them to travel (and thus requiring replacements in clinics and hospitals). It is inherently flexible; as noted previously, it invites further experiments to tweak elements of the intervention itself, find ways of delivering it at lower cost, or both. The present analysis provides details on inputs and costs of our version of mCME, but our findings underscore an important and critical potential to provide effective distance learning to health professionals across Vietnam and other resource-constrained settings at a national scale at low cost. These findings are well-aligned with a similar study in Rwanda that found that a mobile CME app would provide benefits such as rapid decision making, lower error rates, and improve the quality of data management and practice efficiency among clinicians.^5^

Notably, this project was implemented before the coronavirus disease (COVID-19) pandemic, a global phenomenon which has limited in-person activities dramatically around the world. When our team conducted the trial (in a pre-COVID-19 world), we were motivated in part by recent outbreaks such as Zika. Since the cost of setting up the text message platform and enrolling users occurs before delivery of content, we believed that mCME could be a convenient way of delivering novel content in response to new threats, such as Zika or (later) COVID-19. Little did we realize when we launched this work just how relevant it might turn out to be. Indeed, our findings—both the positive effect and the reasonable cost—of the mCME approach only heighten the relevance and potential use of a mobile intervention for critical activities like CME (across many health domains) given the world’s recent COVID-19 pandemic experience.

Our findings on the positive effect and the reasonable cost of the mCME approach only heighten the relevance and potential use of a mobile intervention for critical activities like CME given the recent COVID-19 pandemic.

We note a number of strengths to this analysis. An important strength is that the effect size was measured from an RCT, which enhances the validity and rigor of the findings. That the trial was conducted in 3 provinces across Vietnam—all different geographically and at varying levels of economic development—adds to the reliability and generalizability of the findings. The careful collection of cost data associated with specific activities in the field, rather than relying on abstract estimates or secondhand data from the literature, as many costing studies do, is an important strength of this work. Additionally, we conducted not just a financial cost analysis but also an economic analysis which incorporated the additional labor that was devoted to planning and implementing the intervention beyond what was compensated formally. This component of the analysis strengthens the potential usefulness for policy makers in other settings who want a deep understanding of the resources needed to undertake this type of CME program. Relatedly, the forecasted analyses present 2 cost scenarios for how the mCME intervention may be implemented more broadly in the same population as the one in which the original was executed—providing critical information to inform policy makers’ decision making in Vietnam regarding future CME investments.

### Limitations

We also acknowledge potential limitations of this study. First, our estimates of the cost of the actual mCME intervention include substantial out-of-country financial and economic inputs. For the forecasted models, we assumed task shifting from U.S.-based to local implementation experts that would reduce costs while maintaining effective intervention implementation. Our international team is comfortable with these assumptions given the substantial local technical expertise in Vietnam, but application in other settings may be complicated. Policy makers should bear in mind the technical requirements of a centrally organized text message intervention. A second and related limitation is that the second trial built on technical infrastructure developed for the original mCME trial with community-based providers. This cost was incorporated in the cost analysis of mCME v2.0 but may not have fully captured the time required to establish the initial text message delivery system. Third, our study did not collect actual health outcomes (e.g., improved HIV outcomes) but rather, self-study behaviors and knowledge. We assume these are on the casual pathway between CME and improved patient care but did not confirm that relationship in this study. Fourth, our forecasted model assumed a simple linear series of implementation costs over time after the significant initial investment for technology and infrastructure and did not consider potential variability of costs over time. Lastly, our forecasted analyses did not include projected cost-effectiveness estimates, as we were unable to predict with any degree of confidence future improvements in CME-related behaviors due to the intervention. That said, if even a fraction of the effects of what the mCME trial achieved were to be repeated during a scale up, this would be a remarkable success for CME. Despite these limitations, we believe the aforementioned analyses are informative for programmatic purposes for CME in Vietnam and other similar settings.

## CONCLUSION

This analysis indicates that use of text messages is a feasible and relatively low-cost way to provide effective CME to health professions across Vietnam at a national scale. Additionally, it underscores the way that earlier investments in mobile technology in Vietnam create a foundation on which future low-cost programs may be expanded at scale, with important lessons for other resource-constrained environments. Given the need for feasible, acceptable, and low-cost strategies to deliver CME in resource-constrained regions, this approach is worth further study and possible adoption.

## Notes

**Peer Reviewed**

**First published online:** July 26, 2022.
